# Successful dilation and evacuation for second trimester conjoined twin: a case report and review of the literature

**DOI:** 10.1186/s13256-021-02815-4

**Published:** 2021-05-21

**Authors:** Ferid A. Abubeker, Tesfaye H. Tufa, Matiyas Asrat Shiferaw, Mekdes Daba Feyssa, Wondimu Gudu, Delayehu Bekele, Sarah Prager

**Affiliations:** 1 Department of Obstetrics and Gynecology, Saint Paul’s Hospital Millennium Medical College, Addis Ababa, Ethiopia; 2 Department of Obstetrics and Gynecology, University of Washington, Washington, USA

**Keywords:** Conjoined twins, Dilation and evacuation, Surgical abortion, Second trimester, Case report

## Abstract

**Background:**

Conjoined twins are a rare clinical event occurring in about 1 per 250,000 live births. Though the prognosis of conjoined twins is generally low, there is limited evidence as to the optimal method of pregnancy termination, particularly in cases of advanced gestational age. We report a successful dilation and evacuation (D&E) done for conjoined twins at 22 weeks of gestation.

**Case presentation:**

A 20-year-old primigravid woman was diagnosed with a conjoined, thoraco-omphalopagus twin pregnancy after undergoing a detailed two-dimensional (2D) fetal ultrasound anatomic scanning. Assessment and counseling were done by a multidisciplinary team. The team discussed the prognosis and options of management with the patient. The patient opted for termination of pregnancy. Different options of termination were discussed and the patient consented for D&E, with the possibility of reverting to hysterotomy in case intraoperative difficulty was encountered. A 2-day cervical preparation followed by D&E was done under spinal anesthesia and ultrasound guidance.

**Conclusion:**

In this patient, D&E was done successfully without complications. Adequate cervical preparation, pain control, and ultrasound guidance during the procedure are critical for optimal outcomes. A literature review of methods of pregnancy termination for conjoined twins in the second trimester revealed 75% delivered vaginally through medical induction while 18% underwent cesarean section. Only one other report described successful D&E for conjoined twins after 20 weeks. D&E can be safely performed for carefully selected cases of conjoined twins beyond 20 weeks’ gestations avoiding the need for induction or hysterotomy.

**Supplementary Information:**

The online version contains supplementary material available at https://doi.org/10.1186/s13256-021-02815-4.

## Background

Conjoined twins are extremely rare, occurring in about 1 per 50,000 pregnancies and 1 per 250,000 live births. Though the prognosis of conjoined twins depends on the degree and location of union, it is generally associated with high perinatal mortality and patients may request termination of pregnancy [1, 2]. However, there is limited evidence as to the optimal method of pregnancy termination particularly in cases of advanced gestational age. Though medical terminations of conjoined twin pregnancies have been documented up to late second trimester, the use of surgical methods is not widely reported [3, 4]. Here we report a case of conjoined twins successfully managed with dilation and evacuation (D&E) and systematically review previously reported cases to analyze methods of pregnancy termination for conjoined twins in the second trimester.

## Case presentation

**Fig. 1:**
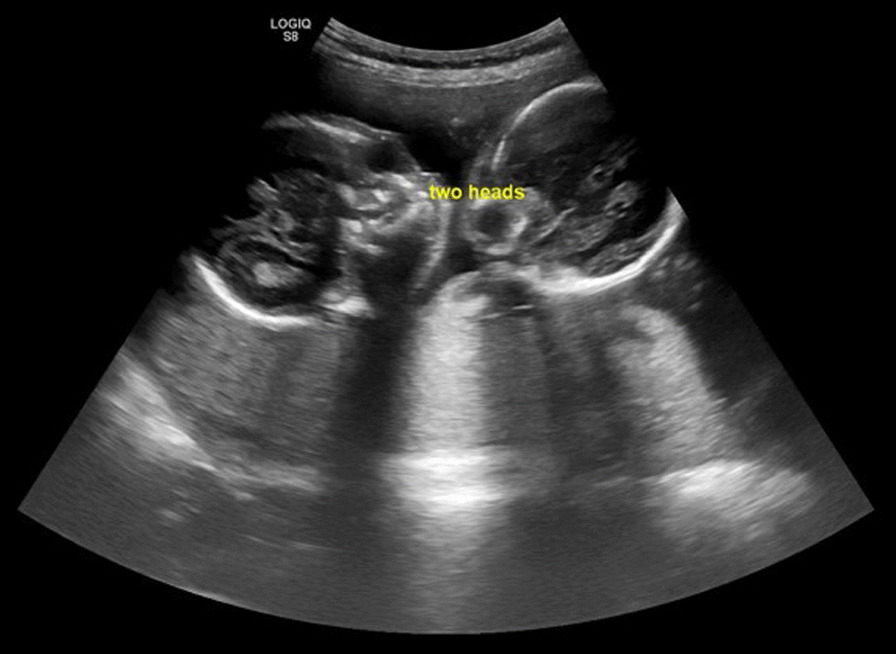
Axial ultrasound image showing two normally shaped fetal heads facing each other

**Fig. 2:**
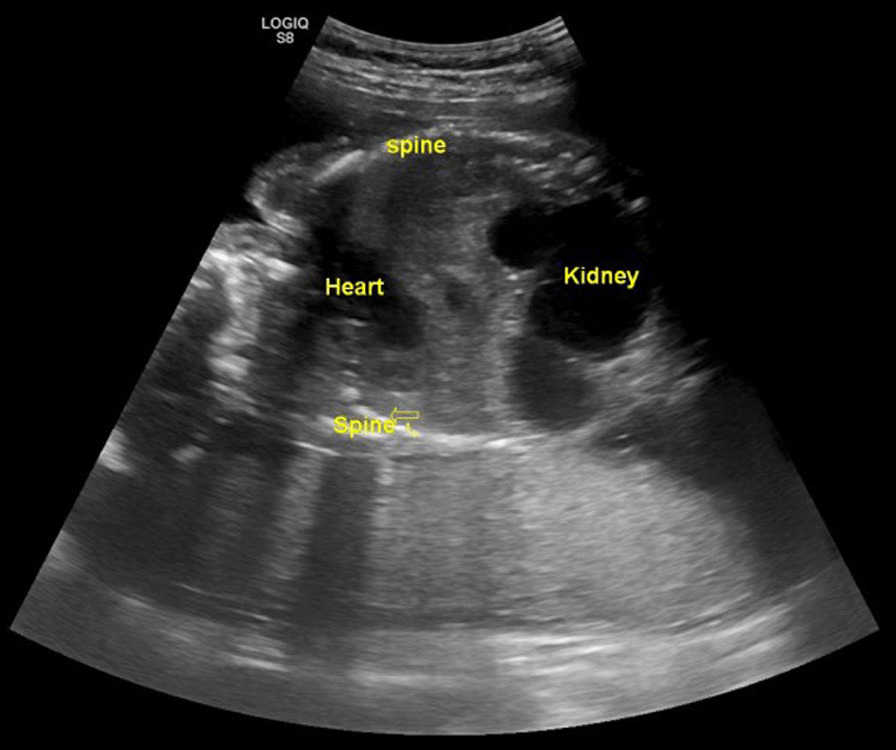
Sagittal section of the fetal chest and abdomen showing two fetal spines, a single distorted heart, and multicystic kidneys

A 20-year-old primigravid woman was referred to our hospital at 22 weeks of gestation with a diagnosis of large fetal intra-abdominal cysts identified during a routine ultrasound examination. In our center, detailed fetal two-dimensional (2D) ultrasound anatomic scanning was done, revealing two fetal heads at a fixed position, facing each other (Fig. 1). There was a fused chest and abdomen with a single shared distorted heart and one aorta. A single umbilical cord was noted. There was a single shared liver. The kidneys appeared enlarged with multiple non-communicating cysts and thinned-out cortical tissue. Two separate spines were visualized on either side of the uterine cavity (Fig. 2). Conjoined, thoraco-omphalopagus twin pregnancy was diagnosed. Fetal karyotyping was offered but declined by the family.

Assessment and counseling were done by a multidisciplinary team composed of obstetricians, fetal medicine specialists, family planning specialists, and anesthetists. After discussion on prognosis and options of management, the patient opted for termination of pregnancy. Different options of termination were discussed and the patient consented for D&E, with the possibility of reverting to hysterotomy in case intraoperative difficulty was encountered.

We performed a 2-day cervical preparation. On day 1, 200 mg mifepristone was administered orally and five laminaria were inserted. On day 2, the patient was admitted and a new set of 10 laminaria were inserted. On the morning of the procedure, she was provided with 400 µg misoprostol sublingually and 200 mg doxycycline orally. After 2 hours she was transferred to the operating room and spinal anesthesia was given. D&E was done under ultrasound guidance. We started the procedure by rupturing the membranes to bring down fetal parts to the lower uterine segment. Initial extraction of fetal parts was done by disarticulating and removing the extremities. Decompression of the thoracic and abdominal cavity allowed further descent and separation of the thoracopagus. The presenting calvarium was decompressed with suction and delivered. Finally, the second twin and placenta were delivered intact. The procedure was completed without complications. Post-procedure tissue count showed two calvaria and spines, four well-formed upper limbs, single thorax and abdomen, and two well-formed and two fused primitive lower limbs. The patient recovered well and was discharged after 24 hours. A follow-up phone call after 2 weeks revealed an uneventful course.

## Discussion

Conjoined twins are rare. The available management options are usually complex and ample experience with case management is limited to few centers worldwide [1]. Recent advances in antenatal imaging techniques, such as three-dimensional (3D) ultrasonography, Doppler studies, and magnetic resonance imaging (MRI), enable diagnosis as early as 12 weeks’ gestation. In addition, detailed prenatal anatomic scanning will define the extent of organ sharing and inform prognosis [2, 5]. Early diagnosis followed by thorough counseling on the likely prognosis is crucial for optimal management [3, 6]. However, as in our case, early diagnosis can be missed and the pregnancy may advance into the second trimester. Other reports from developing countries also show the diagnosis of conjoined twins may be delayed until the third trimester or even up to the time of labor and delivery [7, 8].

Conjoined twins with a shared heart are associated with extremely poor prognosis, and separation and survival of both twins (or even one) is unlikely [2, 9]. Given this, our patient decided to terminate her pregnancy.

Pregnancy termination for conjoined twins in later gestation is often accomplished through hysterotomy because of perceived difficulty in vaginal delivery [10]. Though details of the methods employed were not described, Brizot *et al.* reported 12 vaginal terminations for second trimester conjoined twins [9]. Similarly, Mitchell *et al.* reported two successful inductions of late second trimester conjoined twins. However, both patients underwent two sessions of laminaria placement prior to administration of uterotonics [4].

We conducted a systematic search of the electronic databases of MEDLINE, EMBASE, and Google Scholar using MeSH and keywords from the inception of the databases until November 30, 2020 (see Additional file 1). Bibliographies of the relevant articles were reviewed and then cross-searched to identify further relevant studies. We included all publications in English that specify the method of pregnancy termination for conjoined twins in the second trimester (14–28 weeks).

Two authors (FAA and THT) independently performed study screening and data extraction. Titles and abstracts were screened to identify eligible articles, and full text was obtained if both reviewers judged a citation to be potentially eligible. Standardized screening and data extraction forms were created prior to data collection. Extracted data include author, year of publication, the specific type of conjoined twin, gestational age at termination of pregnancy, method of pregnancy termination, and adverse maternal outcome or procedure-related complications (hemorrhage, blood transfusion, uterine rupture, sepsis, or death). Any discrepancies were resolved through discussion with a third reviewer (MDF).

**Fig. 3:**
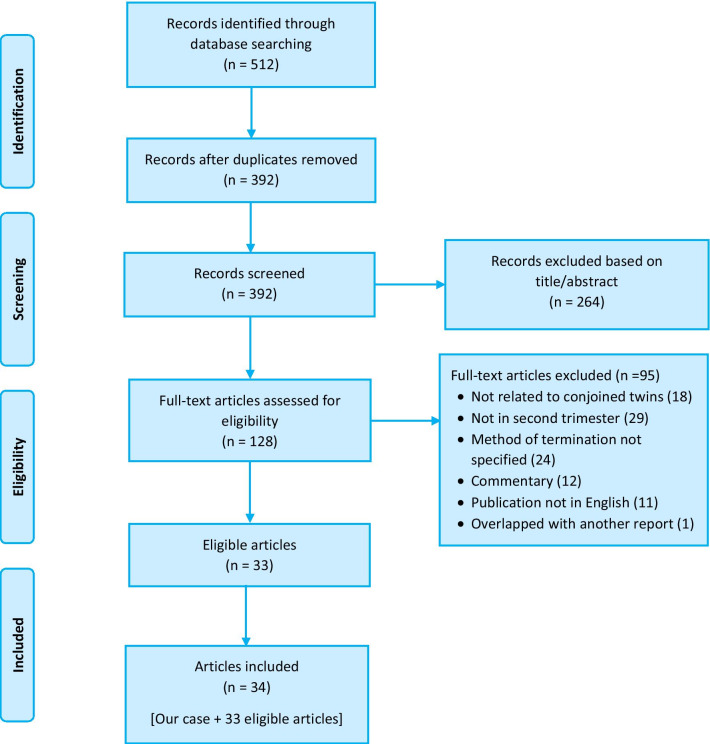
PRISMA flow diagram study screening and selection

Our initial search identified 512 publications. There were 392 articles after duplicates were removed. Examination of title and abstract led to the exclusion of 264 articles. The remaining 128 articles were assessed for eligibility by examining the full text. Of these, 95 were excluded as they did not meet the review inclusion criteria. Thus, our search identified 33 relevant publications with 47 previously reported cases to be eligible. With the addition of the present case, we therefore included a total of 48 cases from 34 publications for this review. Figure 3 presents the PRISMA flow diagram illustrating the systematic selection process.

Most authors resort to medical induction of labor resulting in vaginal delivery; 75% of reviewed cases delivered vaginally through medical induction while 18% underwent cesarean section (Table 1).

**Table 1:** Methods of pregnancy termination for second trimester conjoined twins

	Author, year	Type of union	Gestational age at termination	Method of termination
1	Kattel, 2018 [19]	Parapagus dicephalus	27 weeks 6 days	Cesarean section
2	Sakala, 1986 [10]	Thoracopagus	27 weeks	Vaginal (Pitocin induction)
3	Chatkupt, 1993 [20]	Dicephalus	21 weeks	Vaginal (saline induction)
4	Zoppini, 1993 [21]	Omphalopagus	23 weeks	Cesarean section (classical)
5	Van den Brand, 1994 [22]	Thoracopagus	21 weeks	Both vaginal (prostaglandin induction)
		Omphalopagus	17 weeks	
6	Balakumar, 1995 [12]	Thoraco-omphalopagus	20 weeks	Cesarean section
7	Aquino, 1997 [23]	Craniopagus parasiticus	20 weeks	Vaginal (prostaglandin induction)
8	Sen, 2003 [6]	Thoraco-omphalopagus	19 weeks	Vaginal (misoprostol induction)
9	Esenkaya, 2004 [24]	Dicephalus	17 weeks	Vaginal
10	Tansel, 2004 [25]	Parapagus (dicephalus tetrabrachius dipus)	22 weeks	Vaginal
11	Maymon, 2005 [26]	Thoracopagus	16 weeks	Dilation and evacuation
12	Hassani, 2005 [27]	Dicephalus dibrachius	16 weeks	Vaginal
13	Khanna, 2005 [28]	Cephalothoracopagus janiceps	24 weeks	Vaginal
14	Özkur, 2006 [29]	Cephalopagus	24 weeks	Vaginal (misoprostol induction)
15	Singla, 2009 [11]	Thoracopagus	27 weeks	Vaginal (misoprostol induction)
16	Sabih, 2010 [30]	Dicephalus	24 weeks	Cesarean section
17	Deveer, 2010 [31]	Craniothoracopagus	26 weeks	Vaginal (misoprostol induction)
18	Mete, 2010 [32]	Dicephalic parapagus	16 weeks	Vaginal (misoprostol induction)
19	Camuzcuoglu, 2010 [13]	Dicephalic parapagus	19 weeks	Cesarean section
20	Pandey, 2011 [33]	Thoraco-omphalopagus	15 weeks	Vaginal (misoprostol induction)
21	Brizot, 2011 [9]	A total of 13 cases Thoracopagus 9 Thoracopagus dibrachius tripus 1 Parapagus dibrachius dipus 1 Parapagus dicephalus tribrachius dipus 1 Omphaloischiopagus 1	Gestational age ranging from 18 weeks 3 days to 27 weeks 4 days	One case underwent cesarean section at 27 weeks 4 days. The other 12 had vaginal delivery
22	Pătraşcu, 2013 [34]	Dicephalus dipus dibrachius	21 weeks	Cesarean section
23	Mitchell, 2014 [4]	Thoraco-omphalopagus	23 weeks 6 days	Both vaginal. Inductions were initiated with laminaria and augmented with vaginal misoprostol or oxytocin
		Pygopagus	25 weeks 1 day	
24	Wu, 2014 [35]	Thoracopagus	24 weeks	Vaginal
25	Vaidya, 2014 [36]	Diprosopus	26 weeks	Vaginal (prostaglandin induction)
26	Krawczyk, 2015 [37]	Thoraco-omphalopagus	16 weeks	Vaginal
27	Lu, 2016 [38]	Thoracopagus	25 weeks	Vaginal (prostaglandin induction)
28	Biso, 2017 [39]	Ischiopagus	21 weeks	Vaginal
29	Ozcan, 2017 [14]	Thoraco-omphalopagus	17 weeks	Cesarean section
30	Eris Yalcin, 2018 [40]	Cephalopagus	14 weeks	Vaginal
31	Al Yaqoubi, 2019 [41]	Craniopagus parasiticus	17 weeks	Vaginal (misoprostol induction)
32	Hern, 2019 [15]	Thoracopagus	26 weeks	Dilation and evacuation
33	Vegar-Zubović, 2020 [42]	Cephalothoracoomphalopagus	21 weeks	Cesarean section
34	Our report	Thoracopagus	22 weeks	Dilation and evacuation

Successful induction of labor has been reported for thoracopagus conjoined twins at 27 weeks of gestation [10, 11]. Nevertheless, we identified a few cases of cesarean section performed as early as 20 weeks [12–14].

None of the papers reviewed report adverse maternal outcomes. However, Mitchell *et al.* reported a case complicated by chorioamnionitis. The patient underwent two sessions of laminaria insertion 24 hours apart and was provided with prophylactic antibiotics. Chorioamnionitis was diagnosed on the basis of high-grade fever and tachycardia. She was treated with intravenous antibiotics and was discharged 2 days after successful induction labor [4].

There are limited data on utilization of surgical abortion for conjoined twins. To our knowledge, there is only one report describing successful D&E for conjoined twins after 20 weeks [15]. Although D&E offers a shorter procedure time and avoids the need for induction or hysterotomy, it is not of course without complications, particularly at later gestations. Thus, it should be reserved for specialized centers with experienced providers [3].

When performing D&E, adequate cervical preparation is an important intervention to reduce the risk of procedure-related complications including uterine trauma and cervical laceration. This is especially true in advanced gestational age or, as in our case, when difficulty is anticipated [16, 17]. We achieved adequate cervical preparation with 2 days’ preparation, using a combination of medical and mechanical methods.

The routine use of ultrasound during surgical abortion is controversial. However, ultrasound guidance has been shown to increase safety and facilitate completion of the procedure in difficult cases [18]. We utilized ultrasound throughout the procedure to localize fetal parts and guide our instruments in the uterus.

## Conclusion

Even though this is experience from a single case, D&E can be safely performed for carefully selected cases of conjoined twins beyond 20 weeks’ gestations. Adequate cervical preparation, pain control, and ultrasound guidance during the procedure are critical for optimal outcomes.

## Supplementary Information

**Additional file 1:** Search strategy.

## Data Availability

All data generated or analyzed during this study are included in this published article and its supplementary information files.
