# Supplementary material for: Successful dilation and evacuation for second trimester conjoined twin: a case report and review of the literature
Source: J Med Case Rep. 2021 May 21;15:298. doi: 10.1186/s13256-021-02815-4 (PMC8140421; doi:10.1186/s13256-021-02815-4)
Supplement: Supplementary file 1 — Additional file 1: Search strategy. [file 13256_2021_2815_MOESM1_ESM.docx]

**Search strategy**

| **MEDLINE** | | |
| --- | --- | --- |
| S. No | Concept | Search terms |
|  | Conjoined twin | "Twins, Conjoined"[Mesh] |
|  | Gestational age | "Gestational Age"[Mesh] OR "Pregnancy"[Mesh] OR "Pregnancy Trimester, Second"[Mesh] |
|  | Pregnancy termination and delivery | "Abortion, Induced"[Mesh] OR "Abortion, Therapeutic"[Mesh] OR "Abortion, Legal"[Mesh] OR" Labor, Obstetric/methods"[Mesh] OR "Delivery, Obstetric/methods"[Mesh] OR "Labor, Induced"[Mesh] OR "Misoprostol"[Mesh] OR "Mifepristone"[Mesh] OR "Cesarean Section"[Mesh] OR "Hysterotomy"[Mesh] OR "Dilatation and Curettage"[Mesh] |
| **EMBASE** | | |
| S. No | Concept | Search terms |
|  | Conjoined twin | 'conjoined twins'/exp |
|  | Gestational age | 'gestational age'/exp OR 'second trimester pregnancy'/exp |
|  | Pregnancy termination and delivery | 'induced abortion'/exp OR 'labor induction'/exp OR 'obstetric delivery'/exp OR 'cesarean section'/exp OR 'hysterotomy'/exp OR 'misoprostol'/exp OR 'pregnancy termination'/exp |
| **Google** **Scholar** | | |
| S. No | Concept | Search terms |
|  | Conjoined twin | “Conjoined twins” OR “Thoracopagus” OR “Thoracoomphalopagous” |
|  | Gestational age | “Second trimester” |
|  | Pregnancy termination and delivery | “Dilation and evacuation” OR “Abortion” OR “Pregnancy termination” OR “Induction of labor” OR “Delivery” OR “Cesarean section” |
